# Gain of Chromosome 4qter and Loss of 5pter: An Unusual Case with Features of Cri du Chat Syndrome

**DOI:** 10.1155/2012/153405

**Published:** 2012-12-20

**Authors:** Frenny Sheth, Naresh Gohel, Thomas Liehr, Olakanmi Akinde, Manisha Desai, Olawaleye Adeteye, Jayesh Sheth

**Affiliations:** ^1^FRIGE's Institute of Human Genetics, FRIGE House, Satellite, Ahmedabad 380 015, India; ^2^Critical Neonatal & Child Care Centre, Aakar Complex, Sir T Hospital, Kalanala, Jail Road, Bhavnagar 364 001, India; ^3^Jena University Hospital, Friedrich Schiller University, Institute of Human Genetics, Kollegiengasse 10, 07743 Jena, Germany

## Abstract

Here, we present a case with an unusual chromosomal rearrangement in a child with a predominant phenotype of high-pitched crying showing deletion encompassing *CTNND2* due to an unbalanced translocation of chromosomes 4 and 5. This rearrangement led to a duplication of *~*35 Mb in 4qter which replaced 18 Mb genetic materials in 5pter. Even though, in this patient, there was no clinically obvious modification to the classical phenotypes of CdCS, and the influence of the 4q-duplication cannot be completely excluded in this case. However, the region 4q34.1–34.3 was previously reported as a region not leading to phenotypic changes if present in three copies, an observation which could possibly be supported by this case. *Conclusion*. This study showed that in a patient with an unbalanced translocation resulting in 5p deletion, the presence of partial trisomy of chromosome 4q could be clinically insignificant.

## 1. Introduction

Cri du Chat Syndrome (CdCS) is one of the genetic disorders resulting in form of partial deletions of the short arm of chromosome 5 (5p-). The size of the deletion ranges from the entire short arm to solely 5p15, and all include the *CTNND2* gene. The prevalence is estimated to about 1 in 15,000–50,000 newborns and with females to male ratio of 4 : 3 [1]. Most Cri du Chat Syndrome patients have a *de novo* deletion of about 80%, in which 90% of these are terminal and 10% are interstitial with 80% arising on the paternal homologue. A further 10% arising as a result of a parental translocations and the remainder take place as a result of unusual cytogenetic aberration. Hallmark clinical features of Cri du Chat Syndrome include a high-pitched and monotonous cry, microcephaly, a round face, hypertelorism, epicanthic folds, micrognathia, abnormal dermatoglyphics, growth delay, and severe psychomotor and mental retardation [1].

Although Cri du Chat Syndrome is a well-defined clinical entity, here we report a case in which Cri du Chat Syndrome could not be diagnosed by conventional banding cytogenetic studies alone. Molecular techniques such as fluorescence in situ hybridization (FISH), were applied to confirm the clinical diagnosis and also comprehensively characterize additional genetic material present on the short arm of one chromosome 5.

## 2. Material and Methods

### 2.1. Clinical Description

A 2-month-old male child was born to a healthy and unrelated young couple having a history of two previous first-trimester miscarriages. The child was delivered by caesarean section at the 38th week of gestation. APGAR score was 9. His birth weight was 2,150 g, length was 48 cm, and head circumference was 31 cm.

The neonatal course was complicated by early onset of sepsis and respiratory distress. After 4 days of life, he was presented with feeding problem. Physical examination showed round face, microcephaly, hypertelorism, small eyes, epicanthal fold, small low set ears, and high-pitched cry. Hence, the child was recommended for cytogenetic investigation.

After 30 days of life, he was presented again with failure to thrive, underweight, and bronchiolitis. Clinical examination reveals tachypnoea, hyperbilirubinemia and acyanotic congenital heart defects. The child later expired at the third month of presentation.

### 2.2. Cytogenetic Analysis

**Figure 1:**
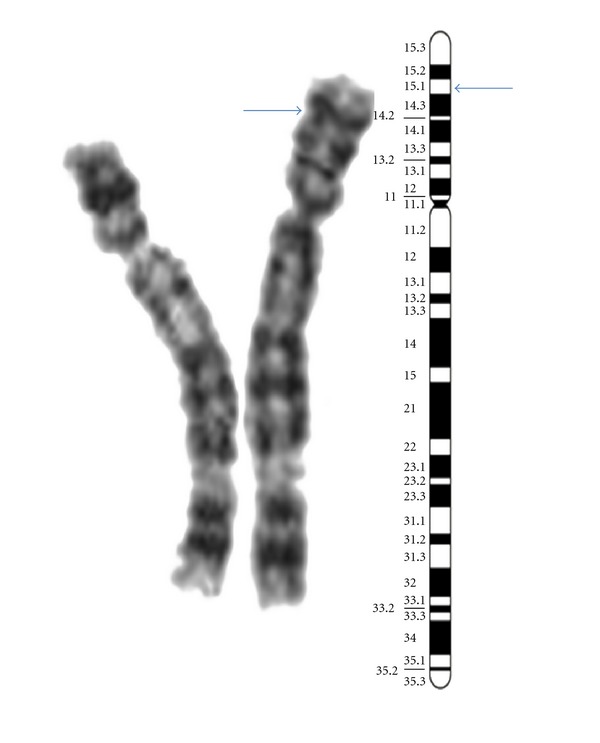
Partial karyotype of patient showing additional material on chromosome 5p as indicated by the arrow.

Cytogenetic study was carried out using peripheral blood lymphocytes for GTG-banding analysis according to the standard procedures. Thirty metaphases were analyzed at 550 bands resolution and karyotyped as per ISCN 2009 [2] guidelines. Metaphase analysis showed additional material of unidentifiable origin at the short arm of one chromosome 5, that is, a karyotype 46,XY,der(5)add(5)(p15;?) (Figure 1). Further molecular cytogenetic characterization of the additional genetic material was carried using FISH. Inheritance could not be established as parents refused to undergo any further investigations.

### 2.3. Molecular Cytogenetic Analysis

FISH was done according to standard procedures using three commercial probes, one for the Cri du Chat Syndrome critical region (LSI Cri du Chat, Abbott/Vysis) and two subtelomeric probes for 5pter and 5qter, respectively, (Abbott, Vysis), as well as the following homemade probes: whole chromosome painting (wcp) probes for all chromosomes used in a multiplex-FISH approach and partial chromosome painting (pcp) probes for 4p and 4q. The homemade probes were produced by chromosome microdissection as described in [3]. Twenty metaphase spreads were analyzed, each using a fluorescence microscope (Axioplan 2 mot, Zeiss) equipped with appropriate filter sets to discriminate between a maximum of five fluorochromes and the counterstain DAPI (Diaminophenylindol). Image capturing and processing were carried out using an mFISH imaging system (MetaSystems, Altlussheim, Germany) for the evaluation MCB.

**Figure 2:**
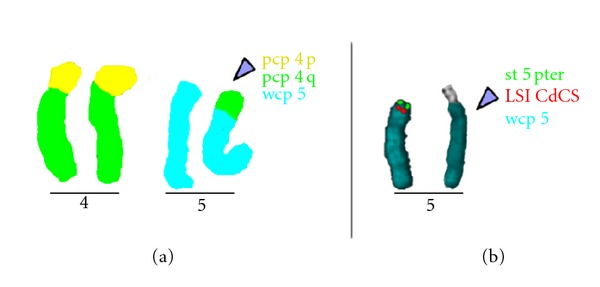
Multiplex-FISH (results were not shown) revealed a chromosome 4 origin of the additional material present on the derivative chromosome 5 (marked with an arrowhead throughout the figure). (a) Three-color FISH using a whole chromosome painting (wcp) probe for chromosome 5 (wcp 5, blue) with a partial chromosome painting (pcp) probe for the long (pcp 4q, green) and the short arm of chromosome 4 (pcp 4p, yellow) highlighting the origin of the additional material on derivative chromosome 5 as 4q derived. (b) A subtelomeric probe for 5pter (st 5pter, green) together with a locus-specific probe for CdCS (red) and a wcp 5 (blue), and the inverted DAPI-banding pattern characterized the breakpoint in chromosome 5 as 5p15.1 and in chromosome 4 as 4q31.3.

After application of the aforementioned probes combined with inverted DAPI-banding (results are shown in Figure 2); the final karyotype could be determined as 46,XY,der(5)t(4;5)(q31.3;p15.1).

## 3. Discussion 

Here, we present an unusual cytogenetic case of a Cri du Chat Syndrome, clinically diagnosed due to the high-pitched cry. Only one allele of the *CTNND2 *gene was present in the genome of the patient. However, the large deletion size together with a partial trisomy of at least 35 megabases (Mb) of 4q31.3 to 4qter as observed here should have resulted in a more severe clinical phenotype in this patient. 

Recurrence risk estimation for the parents of this child is difficult as both parents did not provide consent for further chromosomal analysis. The probability of either parent being a carrier of the balanced chromosomal rearrangements is higher than normal, as they previously had two first-trimester miscarriages. However, the risk of recurrence would be very minimal if the chromosomal imbalance occurred *de novo*.

The predominant phenotypes observed in the present patient was that of Cri du Chat Syndrome despite the extra 4q material [4q31→qter; ~35 Mb] on 5p which replaced the lost [5p15.1→pter; ~18 Mb] genetic material on chromosome 5 short arm. Although Cri du Chat Syndrome is a well-defined clinical entity, individuals with 5p deletion show phenotypic variability. All the phenotypic features present in our patient have been described for Cri du Chat Syndrome in the literature. More severe phenotypes are reported to be associated with large 5p deletions [4]. In patients with an unbalanced translocation resulting in 5p deletion, the partial trisomy of the other involved chromosome did influence the clinical features, even though the Cri du Chat Syndrome phenotype prevailed [1].

Even though, in this patient, there was no clinically obvious modification to the classical phenotypes of Cri du Chat Syndrome; influence of the 4q-duplication on the phenotype has to be taken into account, especially for the observed heart defect [5, 6] for which an association with early death has been seen [6, 7]. Drawback of the present study is that no further molecular genetic investigations were carried out for the 4q duplication that could have probably given an insight into the influence of the genetic alteration on the phenotype. Nonetheless, trisomy of 4q31→4qter may produce symptoms that overlap with that of Cri du Chat that would be masked by the probands underlying condition. Babies and small children with a chromosome disorder appear to have a higher rate of childhood infections involving ears and chest than children with no disorder, and this is true for those with 4q duplication, and our patient was not an exemption of this [8]. The sepsis and respiratory distress he had could have been as a result of pneumonia triggered by recurrent chest infection, poor feeding, and reflux inhalation and complicated with hyperbilirubinemia and failure to thrive.

Overall, the present case of Cri du Chat Syndrome is novel, as the ~18 Mb deletion of 5p material was much more relevant for the phenotype than the ~35 Mb duplication of 4q material. However, this observation supports the hypothesis that in the region 4q34.1–34.3, a 10 Mb stretch was reported as a region not leading to phenotypic changes if present in three copies [9].

## References

[1] Cerruti MP, Pastore G, Castronovo C, Godi M, Guala A, Tamiazzo S (2006). The natural history of Cri du chat syndrome. A report from the Italian register. *European Journal of Medical Genetics*.

[2] Shaffer LG, Slovak ML, Lynda JC (2009). *An International System for Human Cytogenetic Nomenculature*.

[3] Liehr T, Heller A, Starke H (2002). Microdissection based high resolution multicolor banding for all 24 human chromosomes. *International Journal of Molecular Medicine*.

[4] Cerruti PM, Perfumo C, Cali A, Coucourde G, Pastore G, Cavani S (2001). Clinical and molecular characterization of 80 patients with 5p deletions, genotype and phenotype correlation. *Journal of Medical Genetics*.

[5] Elghezal H, Sendi HS, Monastiri K (2004). Large duplication 4q25-q34 with mild clinical effect. *Annales de Genetique*.

[6] Rinaldi R, de Bernardo C, Assumma M (2003). Cytogenetic and molecular characterization of a *de novo* 4q24qter duplication and correlation to the associated phenotype. *American Journal of Medical Genetics A*.

[7] Hubert E, Sawicka A, Wasilewska E, Midro AT (2006). Partial trisomy of long arm of chromosome 4 as a result of dir dup (4)(q27q31.3) *de novo*. *Genetic Counseling*.

[8] Kristensen K, Hjuer T, Ravn H, Simoes EA, Stensballe LG (2012). Chronic diseases, chromosomal abnormalities, and congenital malformations as risks factors for respiratory syncytial virus hospitalization: a population-based cohort study. *Clinical Infectious Diseases*.

[9] Bateman M, Mehta S, Willatt L, Sparnon L, Selkirk E, Bedwell C (2007). A *de novo* 4q34 interstitial deletion of 9–11 Mb with no discernible phenotypic effect. *Journal of Medical Genetics*.

